# Correlation of immune infiltration with clinical outcomes in breast cancer patients: The 25‐gene prognostic signatures model

**DOI:** 10.1002/cam4.3678

**Published:** 2021-02-24

**Authors:** Yushan Liu, Wenfen Fu, Wei Chen, Yuxiang Lin, Jie Zhang, Chuangui Song

**Affiliations:** ^1^ Department of Breast Surgery Fujian Medical University Union Hospital Fuzhou Fujian Province China; ^2^ Department of General Surgery Fujian Medical University Union Hospital Fuzhou Fujian Province China

**Keywords:** breast cancer, cancer genetics, prognosis, TCGA

## Abstract

**Purpose:**

Breast cancer is the most common cancer in women. The aim of this study was to build a prognostic signatures model based on the immune score of the ESTIMATE algorithm to predict survival of breast cancer patients.

**Methods:**

The RNA‐seq expression data and clinical characteristics of patients were derived from TCGA and GSE88770 of GEO. The ESTIMATE algorithm was used to calculate the patients' immune scores and to obtain DEGs. The LASSO Cox regression model was applied to select prognostic genes. Survival analysis and the ROC curve were used to evaluate the predictive efficacy of the prognostic signatures model. Independent prognostic factors of breast cancer were assessed using the Cox regression analyses, and a nomogram was constructed to enhance the clinical value.

**Results:**

Based on the immune score, we found that the high‐score group showed better clinical outcomes than the low‐score group. Twenty‐five (25) genes of 616 DEGs were confirmed as prognostic signatures through the LASSO Cox regression. The risk score for each patient was calculated according to the prognostic signatures. Survival analysis showed that the low‐risk group had longer overall survival than the high‐risk group. We also found that the risk score was an independent prognostic factor. To improve the clinical application value, a nomogram combing the risk score according to the 25‐gene prognostic signatures and several clinicopathological prognostic factors was constructed.

**Conclusions:**

This study revealed the significance of immune infiltration and constructed a 25‐gene prognostic signatures model, that has a strong prognostic value for patients with breast cancer.

## Graphical abstract

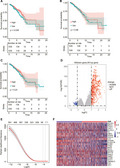

## 1 INTRODUCTION

Breast cancer is the second most common cancer in the world (1.7 million cases, 11.9%), and it is the most frequently seen cancer among women.^1^ Breast cancer is a highly heterogeneous disease, with a slow growth rate, and, while it can be highly invasive, there is a good prognosis for some patients.^2^ At present, the application of surgery, radiotherapy, chemotherapy, hormone therapy, and HER2‐targeted therapy have greatly improved the survival of breast cancer patients. However, some patients still relapsed and have distant metastasis after classical therapies. It remains important to continue looking for novel and effective treatments for breast cancer patients.

With recent developments and applications of new findings in immunology, immunotherapy is coming to play a critical role in cancer treatment. It has become the standard‐of‐care treatment strategy for some malignancies, such as melanoma, lung cancer, and bladder cancer.^3^ In breast cancer, previous studies have confirmed that in patients with early stage triple‐negative and HER2‐positive disease, high levels of lymphocytic infiltration were consistently associated with a better prognosis, and these infiltrations reflect a good host anti‐tumor immune response, suggesting potential benefit of immune activation to improve prognosis.^4^ There have been clinical reports of immune checkpoint blockade monotherapy for several molecular subtypes of breast cancer, and persistent responses have been observed in a significant number of women with chemotherapy‐resistant diseases.^3^ In addition, Atezolizumab (targeted drugs for PD‐L1) in combination with chemotherapy has been recently approved for the treatment of advanced triple‐negative breast cancer.^5^ All these indicate that immunotherapy is expected to achieve encouraging results in the treatment of breast cancer. However, as breast cancer is a heterogeneous disease and most breast cancers exhibit limited innate immunogenicity, it is important to identify relevant biomarkers to distinguish immunotherapy responsive tumors.^3^

In this study, differentially expressed genes (DEGs) were selected based on the immune score using the ESTIMATE algorithm, and a 25‐gene prognostic signatures model was established and validated using The Cancer Genome Atlas (TCGA) and the Gene Expression Omnibus (GEO) databases. We then investigated the survival value of this model, and constructed a more meaningful nomogram combining clinicopathological prognostic factors.

## 2 MATERIALS AND METHODS

### 2.1 Data source

The RNA‐seq expression and clinical characteristic data of breast cancer tumors came from two databases and formed three cohorts. The training cohort was downloaded from TCGA (*n* = 1070) databases (https://portal.gdc.cancer.gov/repository, Table 1). Among patients from the training cohort, we conducted multiple random sampling to establish internal validation cohorts, each containing 100 patients. The external validation cohort data set was the GSE88770 (*n* = 117) from GEO databases (https://www.ncbi.nlm.nih.gov/geo/). Patients with unknown overall survival (OS) were excluded from both data sets.

**TABLE 1:** Clinicopathological characteristics for breast cancer patients in TCGA (*n* = 1070).

Characteristics	*n* (%)
Gender	
Male	12 (1.1)
Female	1058 (98.9)
Status	
Dead	150 (14.0)
Alive	920 (86.0)
Race	
White	746 (69.7)
Asian	58 (5.4)
Other	266 (24.9)
Age^a^	
≤58	546 (51.0)
>58	524 (49.0)
T	
T1	279 (26.1)
T2	617 (57.7)
T3	133 (12.4)
T4	38 (3.6)
TX	3 (0.3)
M	
M0	891 (83.3)
M1	22 (2.1)
MX	157 (14.7)
N	
N0	502 (46.9)
N1	358 (33.5)
N2	120 (11.2)
N3	73 (6.8)
NX	17 (1.6)
TNM stage	
I	181 (16.9)
II	606 (56.6)
III	241 (22.5)
IV	20 (1.9)
X	11 (1.0)
NA	11 (1.0)
PAM50	
Basal‐like	137 (12.8)
HER2‐enrich	62 (5.8)
Luminal A	415 (38.8)
Luminal B	187 (17.5)
Normal‐like	22 (2.1)
NA	247 (23.1)

^a^ The cutoff of age was the median age of TCGA patients with breast cancer; NA means information not available; TCGA, The Cancer Genome Atlas.

### 2.2 ESTIMATE algorithm of breast cancer

The estimate R package was used to implement the ESTIMATE algorithm, which calculated the immune score, stromal score and ESTIMATE score. And patients were divided into two groups, high‐score or low‐score, using the median score of the immune score (stromal score or ESTIMATE score) as the cutoff.

### 2.3 Differential gene expression analysis

The differential gene expression analysis was performed by the limma R package.^6^ The raw data were normalized and transformed to log2‐counts per million (log CPM). The Benjamini‐Hochberg method was adopted to correct the significance *p* value. Finally, the fold change (FC) and the adjusted *p* value (False Discovery Rate, FDR) were adopted as key indexes for screening DEGs. DEGs were selected with |log2(FC)| > 1 and FDR < 0.05.

### 2.4 Construction of the prognostic signatures model

The LASSO Cox regression model was built using the glmnet R package. The optimal λ value was chosen through 10 times cross‐validations. Prognostic signatures were selected and the coefficient of each signature was calculated according to the LASSO Cox regression. The same formula was used to calculate the risk score for each patient. According to the risk score, patients were divided into low‐risk and high‐risk group with the median risk score as the cutoff. Survival analysis and the receiver operating characteristic (ROC) curve were used to test the performance of the prognostic signatures model.

### 2.5 Independent prognosis factors and the nomogram

The Cox regression analyses were used to analysis clinicopathologic characteristics and find independent prognostic factor, *p *< 0.05 was considered statistically significant. To improve the application value, a nomogram was built with the rms R package and its performance was evaluated by C‐index.

### 2.6 Functional enrichment and immune cell infiltration analysis

The Gene Ontology (GO) analysis and the Kyoto Encyclopedia of Genes and Genomes (KEGG) pathway analysis were performed through the clusterProfiler R package.^7^ Biological process, cell component, molecular function and KEGG pathway analysis were performed to enrich the function associated with the DEGs. In addition, Tumor IMmune Estimation Resource (TIMER2.0, http://timer.comp‐genomics.org/) was the online tool that provided us the correlation coefficient of prognostic gene expression and immune cells.^8^ Immune cells included B cells, CD8+T cells, CD4+T cells, macrophages, neutrophils, and dendritic cells. And two of the prognostic genes screened by us, JCHAIN and TMEM273, were not included in the TIMER2.0 analysis.

### 2.7 Statistical analysis

Statistical analyses were conducted with the R program version 4.0.0, using the R studio version software 1.2.5042. The survival R package was used to perform the Kaplan‐Meier survival analysis, the Cox regression analysis and define the C‐index. The survival curve was constructed by the survminer R package. The volcano plot was formed through an R package called ggplot2. The pheatmap R package was used to draw the heatmap. The ROC curve was drawn using the survivalROC R package.

## 3 RESULTS

### 3.1 The acquisition of DEGs based on the immune score

**FIGURE 1:**
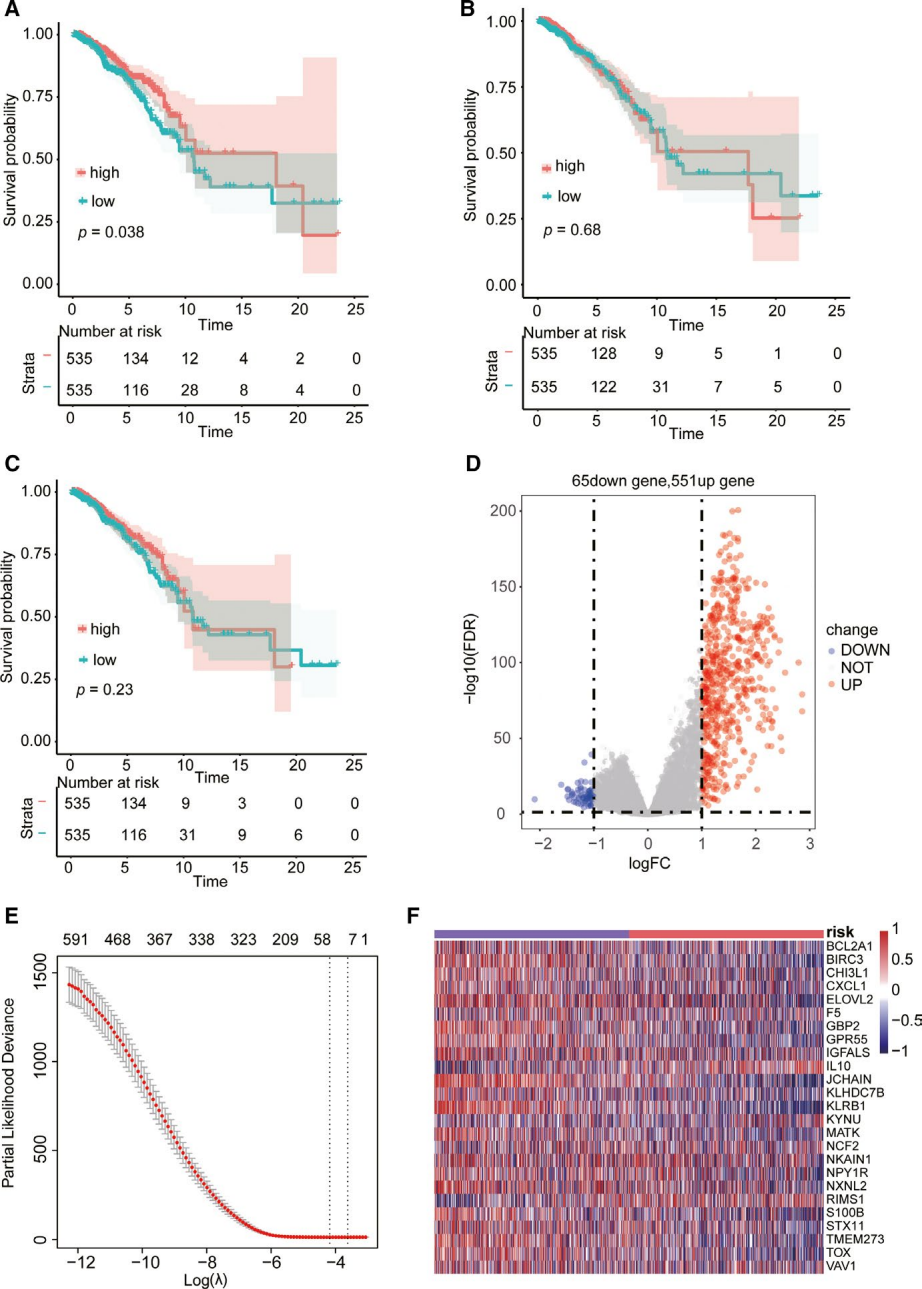
Construction of the 25‐gene prognostic signatures model based on the ESTIMATE algorithm using the LASSO Cox regression model. The OS in the high or low immune score group (A), stromal score group (B), and ESTIMATE score group (C) of patients with breast cancer from TCGA. D, Volcano plot constructed from the immune score shows down‐regulated and up‐regulated DEGs. E, Prognostic signatures were selected using the LASSO Cox regression and the left line shows the optimal values (λ = 0.01534). F, mRNA expression levels in TCGA patients. Red and blue respectively represented high and low risk score group in the risk item (divided by the median risk score).

According to the ESTIMATE algorithm, we obtained the immune score, stromal score, and ESTIMATE score of each breast cancer patient from TCGA. To explore whether a high score was associated with a better prognosis, we performed the survival analysis of two groups that grouped around the median scores. The median immune score was 24.57032, the median stromal score was 404.0199, and the median ESTIMATE score was 465.7355. Based on the immune score, there was a distinct difference between the two groups, and the high‐score group showed longer OS than the low‐score group (*p *= 0.038, Figure 1A). However, in terms of the stromal score, no remarkable difference in survival was found between the two group of patients (*p *= 0.680, Figure 1B). Even though there was no significant difference between the two ESTIMATE score groups, the higher score contributed to a slightly better prognosis (*p *= 0.230, Figure 1C). To further analyze the difference between immune score groups, we compared the high‐score group with the low‐score group by differential gene expression analysis. Finally, we found 616 DEGs that included 65 down‐regulated genes and 551 up‐regulated genes (Figure 1D).

### 3.2 Construction of the 25‐gene prognostic signatures model

To identify potential gene prognostic signatures in DEGs, we built the LASSO Cox regression model, and the value of λ was 0.01534 (Figure 1E). A final prognostic signature model was consisted including 25 genes (Table 2), and the model was used to evaluate the prognosis of the training, external validation, and internal validation cohorts. The mRNA expression levels of these 25 prognostic signatures in TCGA is shown in Figure 1F.

**TABLE 2:** Information of the 25‐gene prognostic signatures.

Gene	Full name	Coefficient
BCL2A1	B‐cell Lymphoma 2‐Related Protein Al	−0.094812912
BIRC3	Baculoviral IAP Repeat Containing 3	−0.011143758
CHI3L1	Chitinase 3 Like 1	−0.00455589
CXCL1	C‐X‐C Motif Chemokine Ligand 1	−0.007613345
EL0VL2	Elongation of Very Long‐Chain Fatty Acids Elongase 2	−0.041806646
F5	Coagulation Factor V	−0.020145838
GBP2	Guanylate Binding Protein 2	−0.052726578
GPR55	G Protein‐Coupled Receptor 55	−0.013097709
IGFALS	Insulin Like Growth Factor Binding Protein Acid Labile Subunit	−0.019624008
IL10	Interleukin 10	0.258800653
JCHAIN	Joining Chain of Multimeric IgA And IgM	−0.046765922
KLHDC7B	Kelch Domain Containing 7B	−0.057177035
KLRB1	Killer Cell Lectin Like Receptor Bl	−0.198897444
KYNU	Kynureninase	0.025439689
MATK	Megakaryocyte‐Associated Tyrosine Kinase	−0.038183565
NCF2	Neutrophil Cytosolic Factor 2	−0.004022496
NKAIN1	Sodium/Potassium Transporting ATPase Interacting 1	−0.025904294
NPY1R	Neuropeptide Y Receptor Y1	−0.002076418
NXNL2	Nucleoredoxin Like 2	−0.042055956
RIMS1	Regulating Synaptic Membrane Exocytosis 1	0.010435445
S1OOB	S100 Calcium Binding Protein B	−0.005558833
STX11	Syntaxin 11	−0.048805878
TMEM273	Transmembrane Protein 273	0.139700396
TOX	Thymocyte Selection Associated High Mobility Group Box	−0.004077689
VAV1	Vav Guanine Nucleotide Exchange Factor 1	0.058218322

### 3.3 Survival validation of the 25‐gene prognostic signatures model and subgroup analysis

**FIGURE 2:**
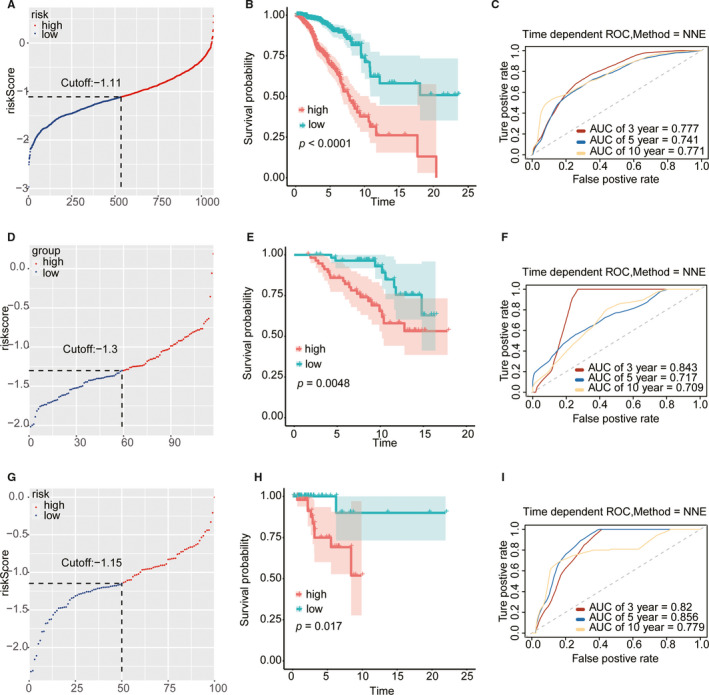
Survival verification of the risk score. The risk score distribution of the training cohort (A), the external validation cohort (D) and the internal validation cohort (G). The Survival curves of the training cohort (B), the external validation cohort (E), and the internal validation cohort (H). The ROC curves for 3, 5, and 10 years survival of the training cohort (C), the external validation cohort (F), and the internal validation cohort (I).

**FIGURE 3:**
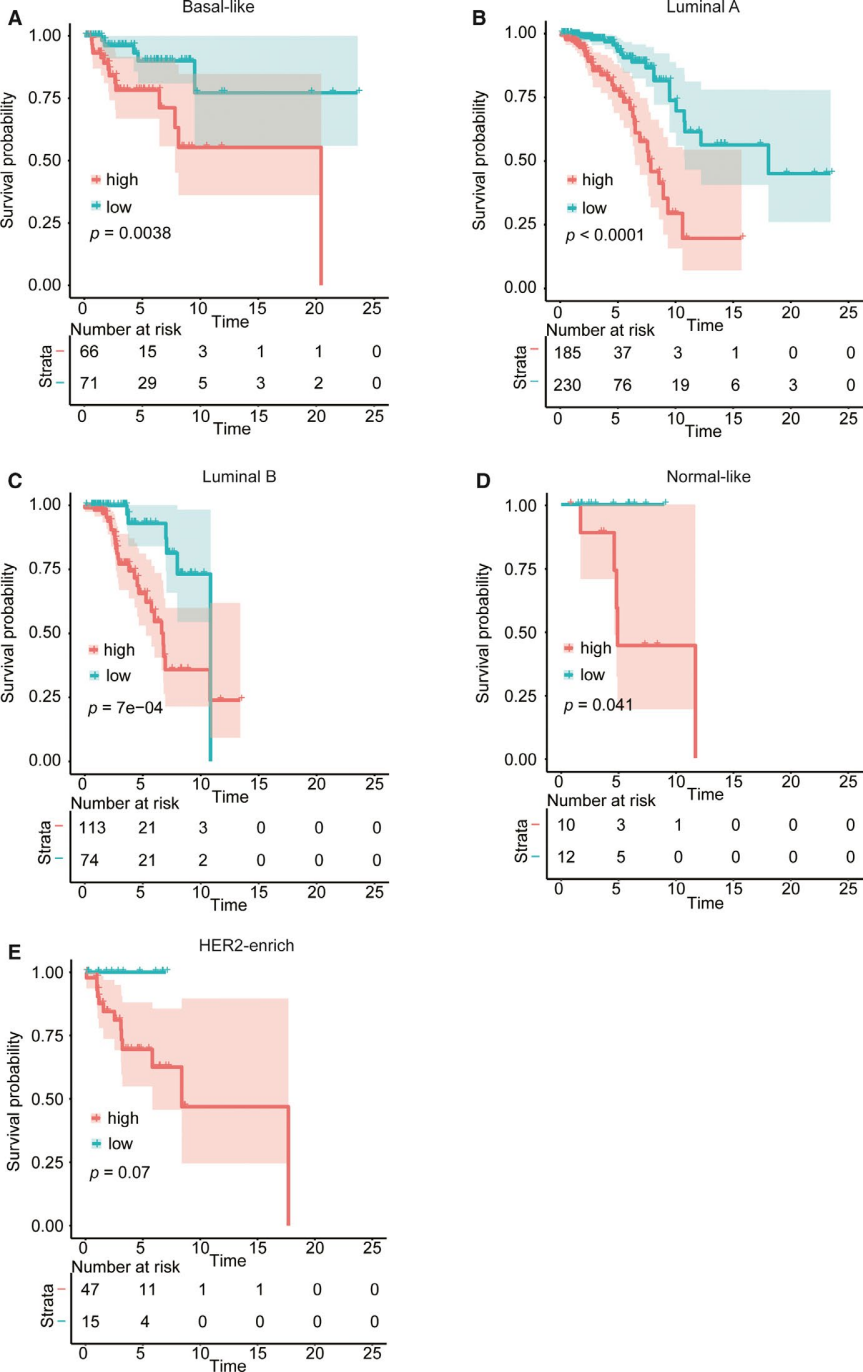
Kaplan‐Meier survival analysis of different PAM50 subtypes in TCGA. The subtypes included Basal‐like (A), Luminal A (B), Luminal B (C), Normal‐like (D), and HER2‐enrich (E) breast cancer. The *p* < 0.05 was considered statistically significant.

Based on the 25‐gene prognostic signatures model, we calculated each patient's risk score and grouped them according to the median risk score. In the training cohort (TCGA), there was a significant difference between the OS of the low‐risk and high‐risk score group, and the low‐risk group demonstrated a longer OS. Furthermore, similar to the training cohort, lower scores were associated with better prognosis in the external (GSE88770) and internal validation cohorts. To further evaluate the survival validation of the model, we constructed ROC curves. In the training, external validation and one of the internal validation cohorts, the area under the curve (AUC) values of the 3‐year cohort were 0.777, 0.843, and 0.820, the 5‐year AUC cohort values were 0.741, 0.717, and 0.856, and the 10‐year AUC cohort values were 0.771, 0.709, and 0.779, respectively. (Figure 2A‐I, Table S1). In addition, the subgroups analysis of patients with different PAM50 subtypes in TCGA indicated that a lower risk score predicted better OS. Good predicting function was found in the Basal‐like (*p* = 0.0038), Luminal A (*p* < 0.0001), Luminal B (*p* = 7e‐04) and Normal‐like (*p* = 0.041) breast cancer patients. Although there were no significant differences between the two groups of the HER2‐enrich subtype (*p* = 0.070), the low‐risk score group still had a better OS than the high‐risk score group (Figure 3A‐E).

### 3.4 A nomogram with better predictive value

**FIGURE 4:**
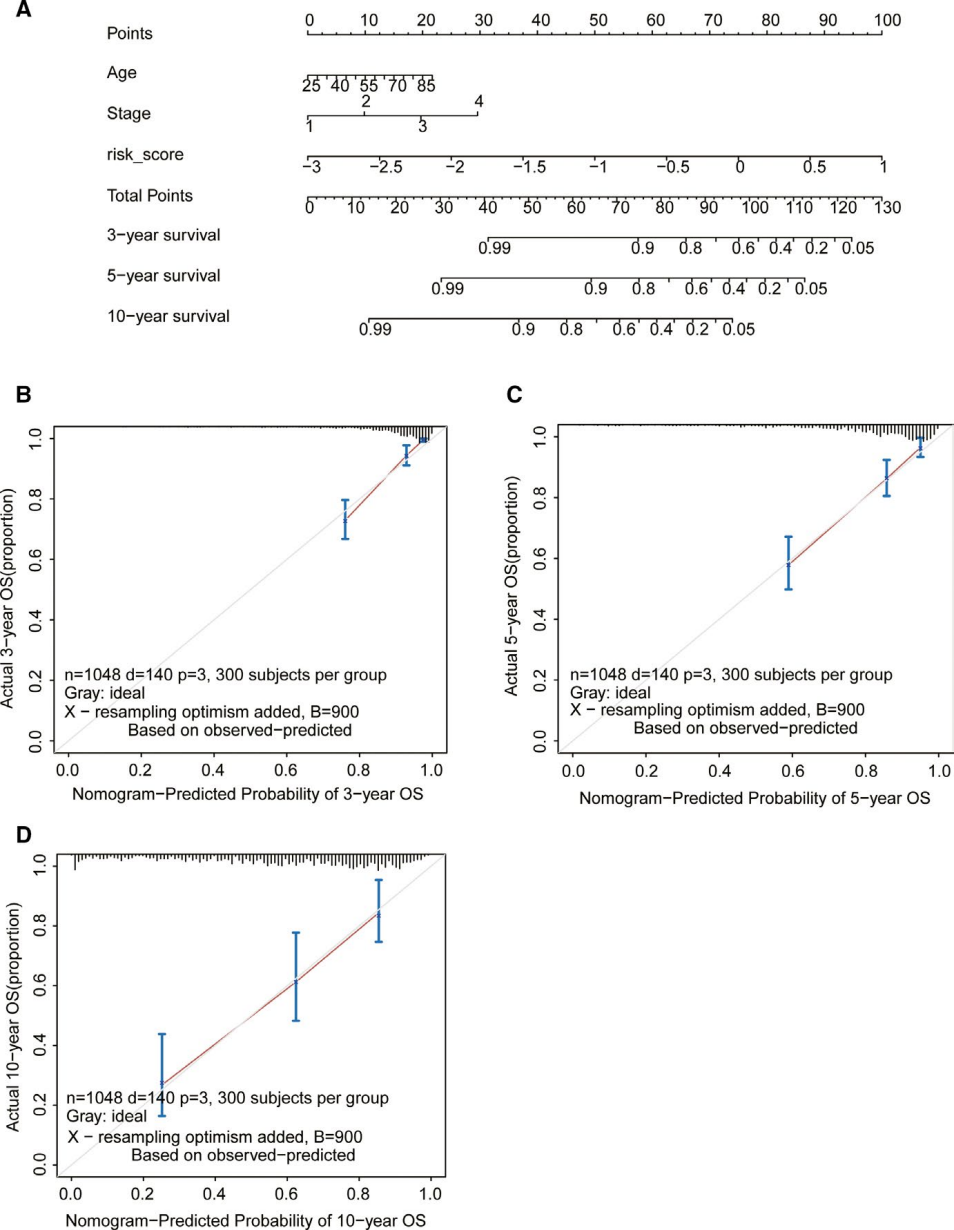
The nomogram combining the risk score and clinicopathological prognostic factors from breast cancer patients from TCGA. A, A nomogram to predict 3, 5, and 10 years survival for breast cancer patients. Calibration curves of the nomogram for 3 (B), 5 (C), and 10 (D) years survival.

To determine whether the risk stratification formed by the 25‐gene prognostic signatures model is an independent prognostic determinant, and to look for other clinicopathological independent prognostic factors, we performed univariate and multivariate Cox regression analyses in the TCGA cohort. The clinicopathological factors included gender, age, primary site of tumor (T), regional lymph node (N), metastasis (M), and TNM stage (Table 3). We confirmed that age, TNM stage and the risk score were independent prognostic factors for breast cancer. To enhance the clinical value of the 25‐gene prognostic signatures model, we constructed a nomogram combining the risk score and clinicopathological independent prognostic factors. The nomogram was used to predict patient OS at 3‐year, 5‐year, and 10‐year. We found that a lower score means a longer OS (Figure 4A). The 3‐year, 5‐year, and 10‐year calibration curves coincided well with the median line (Figure 4B‐D), and the C‐index of the nomogram was 0.82824083. This indicates that the nomogram has a strong predictive value for patient OS. What is more, compared to the 25‐gene prognostic signatures model or other clinicopathological independent prognostic factors, this nomogram was a better predictor (Table 4).

**TABLE 3:** Univariate and Multivariate Cox regression analyses in TCGA.

	Univariate			Multivariate		
Characteristics	HR	CI 95	*p* value	HR	CI 95	*p* value
Gender (male vs female)	1.21	0.17–8.68	0.848			
Age (≤58 vs >58)	1.75	1.27–2.42	0.001	1.58	1.1–2.28	0.014
T(T1‐2vs3‐4)	1.76	1.22–2.53	0.002	0.95	0.58–1.55	0.841
N (N0 vs N1‐3)	2.2	1.54–3.14	0.000*	1.51	0.97–2.35	0.069
M (M0 vs M1)	4.88	2.91–8.17	0.000*	1.83	0.94–3.55	0.075
TNM stage (I‐II vs lll‐IV)	2.62	1.87–3.66	0.000*	1.83	1.08–3.12	0.025
Risk score (low vs high)	4.22	2.94–6.07	0.000*	4.07	2.7–6.13	0.000*

HR, Hazard Ratio; CI 95, 95% confidence interval.

* *p* < 0.001.

**TABLE 4:** C‐index analysis of models.

Model	C‐index
Age	0.63787373
TNM stage	0.68429704
25‐gene prognostic signatures	0.77813022
Nomogram	0.82824083

### 3.5 Functional enrichment analysis correlated with DEGs

**FIGURE 5:**
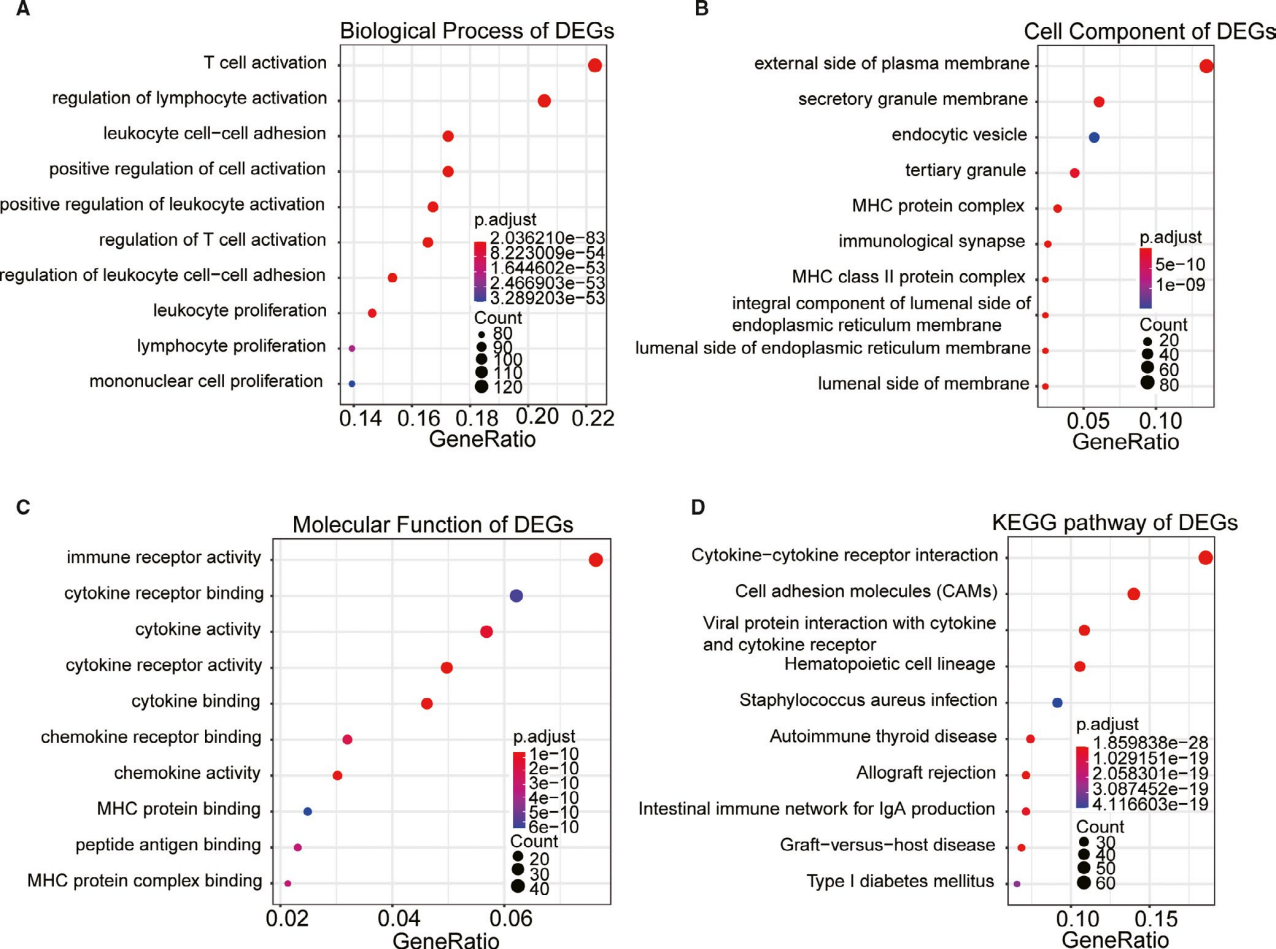
Functional enrichment analysis for DEGs. The top 10 terms among the biological process (A), the cell component (B), the molecular function (C), and the KEGG pathway (D).

To further explore the function of DEGs, we conducted GO analysis and KEGG pathway analysis. We listed the top 10 terms among biological processes, cell components, molecular functions and KEGG pathway (Figure 5A‐D). We found that biological processes of DEGs were significantly associated with an immune response, such as T cell activation, regulation of lymphocyte activation, and lymphocyte proliferation. In addition, there was also a degree of immune correlation in the cellular component, molecular function and KEGG pathway, including immunological synapse, immune receptor activity, cytokine−cytokine receptor interaction and so on.

### 3.6 Association between prognostic signatures and tumor immune infiltrating cells

**FIGURE 6:**
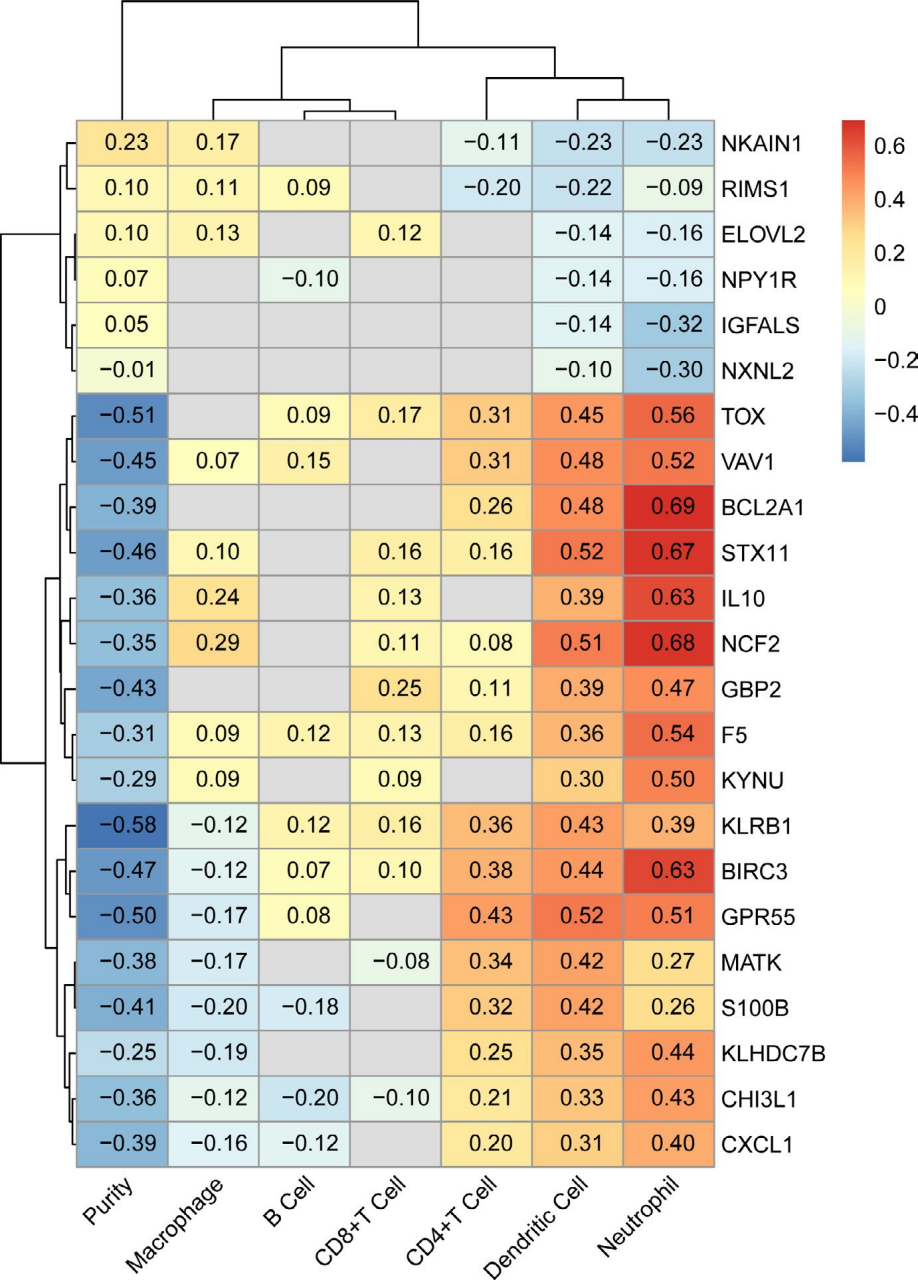
Relationship between prognostic signatures and immune infiltrating cells. The color represented the degree of correlation, red mean a positive correlation, blue mean a negative correlation, and gray mean no statistical significance (*p* > 0.05). The number in the grid is the correlation coefficient.

We used TIMER2.0 to predict the relationship between expression of prognostic signatures and six types of immune cells. The heatmap shows that TOX, VAV1, BCL2A1, STX11, IL10, NCF2, GBP2, F5, and KYNU were strongly correlated with six kinds of immune cells including macrophages, B cells, CD8+T cells, CD4+T cells, dendritic cells and neutrophils, especially dendritic cells and neutrophils. In contrast, some genes had limited connections to immune cells, including NKAIN1, RIMS1, ELOVL2, NPY1R, IGFALS, and NXNL2. Moreover KLRB1, BIRC3, GPR55, MATK, S100B, KLHDC7B, CHI3L1, and CXCL1 were associated with certain immune cells, such as CD4+T cells, neutrophils, and dendritic cells. But in other immune cells, such as macrophage, there was a lower correlation. (Figure 6).

## 4 DISCUSSION

The tumor microenvironment is extremely complex, including not only epithelial cells, vascular cells, stromal cells, but also a large number of infiltrating immune cells. Infiltrating immune cells, which function in an environmentally dependent manner, have been shown to play an antitumor role in some tumors and are closely related to tumor growth, invasion, and metastasis.^9^ In breast cancer, many studies have been conducted in terms of the correlation between the tumor immune cell infiltration and clinical outcome, and it has been reported that high levels of immune cell infiltration are associated with better outcomes.^10^, ^11^, ^12^ In addition, the infiltration of lymphocytes in breast cancers is highly correlated with the sensitivity to chemotherapy.^13^ Therefore, here we focused on the infiltration immune cells to build a prognostic model for patients with breast cancer.

The ESTIMATE algorithm is a way to use gene expression characteristics to infer the proportion of stromal cells and immune cells in a tumor sample. Based on the immune signature (141 genes) and stromal signature (141 genes), the immune score and stromal score were obtained for each sample to predict the infiltration of stromal and immune cells in tumor tissues. And the ESTIMATE score, that combined immune and stromal scores, was calculated to infer tumor purity in tumor tissue. The predictive power of the ESTIMATE algorithm has been demonstrated in a variety of tumors.^14^

Our study focused on the immune score of the ESTIMATE algorithm. The TCGA cases were grouped according to their immune scores, and the results showed that patients with high scores had better OS compared to those with the low scores. In addition, the related DEGs were obtained. The 25‐gene prognostic signatures model was constructed with the LASSO Cox regression model, and the risk score calculated by this prognostic model was negatively correlated with survival. Furthermore, we found that the risk score could be used as an independent prognostic factor for breast cancer patients.

Existing studies have confirmed an important role of immune cells including T lymphocytes, macrophages, and dendritic cells in the growth, progression, prognosis, and treatment of breast cancer patients.^15^, ^16^, ^17^, ^18^, ^19^, ^20^, ^21^ Therefore, through TIMER2.0, we identified the correlation between prognostic signatures and tumor immune cell infiltration, and the result was consistent with the regulation of DEGs. Compared with the low immune score group, TOX, VAV1, BCL2A1, STX11, IL10, NCF2, GBP2, F5, KYNU, KLRB1, BIRC3, GPR55, MATK, S100B, KLHDC7B, CHI3L1, and CXCL1 were up‐regulated genes in the high immune score group, and had a better correlation with immune cells. On the contrary, NKAIN1, RIMS1, ELOVL2, NPY1R, IGFALS, and NXNL2 were all down‐regulated genes and poorly correlated with immune cells.

Some of the prognostic signatures that showed a remarkable correlation coefficient with immune cells have been shown in prior studies to play an important role not only in the occurrence and development of tumors, but also in the regulation of the immune system. BCL2A1 is an apoptosis modulator that is overexpressed as a nuclear factor kappa B target gene in many cancer cells and contributes to tumor progression. Combined with the results of our analysis (Figure 6), BCL2A1 showed a chemotactic role for T lymphocytes, dendritic cells, and neutrophils. In fact, BCL2A1 plays a crucial role in lymphocyte development, mast cell mediated allergic reactions, and lymphocyte and macrophage activation, especially the up‐regulation of BCL2A1 to regulate the CD40 survival signaling pathway of B lymphocytes.^22^, ^23^ BIRC3 is a multifunctional protein that regulates immunity, apoptosis, metastasis, and other functions. It not only has an obvious chemotaxis effect on immune cells, such as B lymphocytes, T lymphocytes, and neutrophils, but also participates in the TNFR2/BIRC3‐TRAF1 signaling pathway, that is a novel NK cell immune checkpoint for cancer.^24^ Il‐10 is a cytokine that induces an immune response and an anti‐inflammatory microenvironment that promotes tumor growth by helping tumor cells evade immune surveillance. It can be used as an indicator to evaluate the efficacy of neoadjuvant chemotherapy in patients with HER2‐enrich breast cancer.^25^ The CHI3L1 gene product is a glycoprotein that can be expressed and secreted by both tumor cells and immune cells. CHI3L1 can promote tumor progression by upregulation of pro‐inflammatory mediators, like CCL2, CXCL2, and MMP‐9. Tumor‐recruited M2 macrophages can secret CHI3L1 to promote the metastasis of gastric cancer and breast cancer, and elevated serum levels of CHI3L1 glycoprotein are associated with poor prognosis in patients with metastatic breast cancer.^26^, ^27^ CXCL1 is significantly correlated with T lymphocyte infiltration, during the progression of breast cancer CXCL1 is up‐regulated by Th17 cells and can promote the growth and development of breast cancer.^28^ GBP2 contributes to better clinical outcomes in rapidly proliferating breast tumors and may serve as a marker for an effective T cell response.^29^ TOX is closely related to depletion of CD8(+) T cells. It is expressed in most circulating effector cell memory CD8(+) T cell subsets, and the knockout of TOX in human tumor‐infiltrating CD8(+) T cells can lead to down‐regulation of PD‐1, TIM‐3, TIGIT, and CTLA‐4, suggesting that the TOX can promote T cell failure by up‐regulating immune checkpoint proteins in tumors. In addition, TOX expression in tumor‐infiltrating T cells can be used to stratify patients during antitumor therapy, including anti‐PD‐1 immunotherapy.^30^, ^31^ Other genes in our model are also associated with physiological and pathological processes in tumors.

According to our study, there are unique correlations between various genetic prognostic signatures and types and numbers of immune cells. This may indicate the unique functions of these genes in the tumor immune microenvironment, suggesting that immune infiltration may influence the occurrence and development of tumors through specific pathways. Consideration of these results may provide new directions for immunotherapy of breast cancer patients.

Of many prognostic indicators of breast cancer have been reported, the Oncotype DX Recurrence Score is the most widely used test to clinically assess the risk of recurrence in patients receiving endocrine therapy.^32^ Similar tests include Prosigna,^33^ Breast Cancer Index,^34^ and EndoPredict.^35^ In addition, MammaPrint has been shown to improve the prediction of clinical outcomes in patients with early breast cancer.^36^ These indicators are more limited to a certain subtype or stage of breast cancer and are used to screen patients who can be exempted from chemotherapy. However, the data of our prediction model is based on the total population of breast cancer patients, from which the high‐risk population is screened. The selection of prognostic signatures is closely related to immune infiltration, which is helpful to find the target of immunotherapy.

We have identified the following limitations of this study. First of all, our study was mainly based on the overall data of TCGA for breast cancer, although it had an optimistic predictive effect in most molecular subtypes of breast cancer, its predictive effect in breast cancer subtypes needed to be confirmed by increasing the sample size of each subtype. Second, the study was performed as a retrospective analysis, and prospective cohort studies are required to verify our findings. Furthermore, our study needs to be validated by clinical specimens and we will further explore it in our future work. Finally, in the process of clinical application, choosing an appropriate cutoff of risk score is a problem that needs to be further discussed.

In summary, our study constructed a 25‐gene prognostic signatures model that associated with immune infiltration. And it can be used as an independent prognostic factor for predicting clinical outcomes in patients with breast cancer.

## 5 ETHICS APPROVAL AND INFORMED CONSENT

This study was approved by Ethics Committee of Fujian Medical University Union Hospital and in accordance with the Declaration Helsinki and its later amendments or comparable ethical standards. All data in this study were obtained from published researches which contained informed consent.

## CONFLICTS OF INTEREST

The authors declare that the research was conducted in the absence of any commercial or financial relationships that could be construed as a potential conflict of interest.

## AUTHOR CONTRIBUTIONS

YL and WF designed the study. YL, WF, and WC obtained and analyzed the data. YL, WF, YXL, JZ, and CS unscrambled the statistical result. YL and WF wrote the manuscript. All authors reviewed and modified the manuscript.

## Supporting information

Table S1

## Data Availability

The data in this study was obtained from the TCGA databases (https://portal.gdc.cancer.gov/repository) and the GEO databases (https://www.ncbi.nlm.nih.gov/geo/).

## References

[1] Ferlay J , Soerjomataram I , Dikshit R , et al. Cancer incidence and mortality worldwide: sources, methods and major patterns in GLOBOCAN 2012. Int J Cancer. 2015;136(5):E359‐E386. 25220842 10.1002/ijc.29210

[2] Verma R , Bowen RL , Slater SE , Mihaimeed F , Jones JL . Pathological and epidemiological factors associated with advanced stage at diagnosis of breast cancer. Br Med Bull. 2012;103(1):129‐145. 22864058 10.1093/bmb/lds018

[3] Sanchez K , Page D , McArthur HL . Immunotherapy in breast cancer: an overview of modern checkpoint blockade strategies and vaccines. Curr Probl Cancer. 2016;40(2–4):151‐162. 27855963 10.1016/j.currproblcancer.2016.09.009

[4] Savas P , Salgado R , Denkert C , et al. Clinical relevance of host immunity in breast cancer: from TILs to the clinic. Nat Rev Clin Oncol. 2016;13(4):228‐241. 26667975 10.1038/nrclinonc.2015.215

[5] Schmid P , Adams S , Rugo HS , et al. Atezolizumab and Nab‐paclitaxel in advanced triple‐negative breast cancer. N Engl J Med. 2018;379(22):2108‐2121. 30345906 10.1056/NEJMoa1809615

[6] Ritchie ME , Phipson B , Wu D , et al. limma powers differential expression analyses for RNA‐sequencing and microarray studies. Nucleic Acids Res. 2015;43(7):e47. 25605792 10.1093/nar/gkv007 PMC4402510

[7] Yu G , Wang LG , Han Y , He QY . clusterProfiler: an R package for comparing biological themes among gene clusters. OMICS. 2012;16(5):284‐287. 22455463 10.1089/omi.2011.0118 PMC3339379

[8] Li T , Fu J , Zeng Z , et al. TIMER2.0 for analysis of tumor‐infiltrating immune cells. Nucleic Acids Res. 2020;48(W1):W509‐W514. 32442275 10.1093/nar/gkaa407 PMC7319575

[9] Fridman WH , Pages F , Sautes‐Fridman C , Galon J . The immune contexture in human tumours: impact on clinical outcome. Nat Rev Cancer. 2012;12(4):298‐306. 22419253 10.1038/nrc3245

[10] Yoon NK , Maresh EL , Shen D , et al. Higher levels of GATA3 predict better survival in women with breast cancer. Hum Pathol. 2010;41(12):1794‐1801. 21078439 10.1016/j.humpath.2010.06.010 PMC2983489

[11] Oldford SA , Robb JD , Codner D , Gadag V , Watson PH , Drover S . Tumor cell expression of HLA‐DM associates with a Th1 profile and predicts improved survival in breast carcinoma patients. Int Immunol. 2006;18(11):1591‐1602. 16987935 10.1093/intimm/dxl092

[12] Teschendorff AE , Gomez S , Arenas A , et al. Improved prognostic classification of breast cancer defined by antagonistic activation patterns of immune response pathway modules. BMC Cancer. 2010;10:604. 21050467 10.1186/1471-2407-10-604 PMC2991308

[13] Andre F , Berrada N , Desmedt C . Implication of tumor microenvironment in the resistance to chemotherapy in breast cancer patients. Curr Opin Oncol. 2010;22(6):547‐551. 20842030 10.1097/CCO.0b013e32833fb384

[14] Yoshihara K , Shahmoradgoli M , Martinez E , et al. Inferring tumour purity and stromal and immune cell admixture from expression data. Nat Commun. 2013;4:2612. 24113773 10.1038/ncomms3612 PMC3826632

[15] Queen MM , Ryan RE , Holzer RG , Keller‐Peck CR , Jorcyk CL . Breast cancer cells stimulate neutrophils to produce oncostatin M: potential implications for tumor progression. Cancer Res. 2005;65(19):8896‐8904. 16204061 10.1158/0008-5472.CAN-05-1734

[16] Bates GJ , Fox SB , Han C , et al. Quantification of regulatory T cells enables the identification of high‐risk breast cancer patients and those at risk of late relapse. J Clin Oncol. 2006;24(34):5373‐5380. 17135638 10.1200/JCO.2006.05.9584

[17] Mahmoud SM , Paish EC , Powe DG , et al. Tumor‐infiltrating CD8+ lymphocytes predict clinical outcome in breast cancer. J Clin Oncol. 2011;29(15):1949‐1955. 21483002 10.1200/JCO.2010.30.5037

[18] Ali HR , Provenzano E , Dawson SJ , et al. Association between CD8+ T‐cell infiltration and breast cancer survival in 12,439 patients. Ann Oncol. 2014;25(8):1536‐1543. 24915873 10.1093/annonc/mdu191

[19] Su S , Liao J , Liu J , et al. Blocking the recruitment of naive CD4(+) T cells reverses immunosuppression in breast cancer. Cell Res. 2017;27(4):461‐482. 28290464 10.1038/cr.2017.34 PMC5385617

[20] Qiu SQ , Waaijer SJH , Zwager MC , de Vries EGE , van der Vegt B , Schröder CP . Tumor‐associated macrophages in breast cancer: Innocent bystander or important player? Cancer Treat Rev. 2018;70:178‐189. 30227299 10.1016/j.ctrv.2018.08.010

[21] Schmidt M , Weyer‐Elberich V , Hengstler JG , et al. Prognostic impact of CD4‐positive T cell subsets in early breast cancer: a study based on the FinHer trial patient population. Breast Cancer Res. 2018;20(1):15. 29482642 10.1186/s13058-018-0942-x PMC5827982

[22] Vogler M . BCL2A1: the underdog in the BCL2 family. Cell Death Differ. 2012;19(1):67‐74. 22075983 10.1038/cdd.2011.158 PMC3252829

[23] Lee HH , Dadgostar H , Cheng Q , Shu J , Cheng G . NF‐kappaB‐mediated up‐regulation of Bcl‐x and Bfl‐1/A1 is required for CD40 survival signaling in B lymphocytes. Proc Natl Acad Sci USA. 1999;96(16):9136‐9141. 10430908 10.1073/pnas.96.16.9136 PMC17745

[24] Ivagnes A , Messaoudene M , Stoll G , et al. TNFR2/BIRC3‐TRAF1 signaling pathway as a novel NK cell immune checkpoint in cancer. Oncoimmunology. 2018;7(12):e1386826. 30524877 10.1080/2162402X.2017.1386826 PMC6279330

[25] Valdes‐Ferrada J , Munoz‐Durango N , Perez‐Sepulveda A , et al. Peripheral blood classical monocytes and plasma interleukin 10 are associated to neoadjuvant chemotherapy response in breast cancer patients. Front Immunol. 2020;11:1413. 32733470 10.3389/fimmu.2020.01413 PMC7363840

[26] Chen Y , Zhang S , Wang Q , Zhang X . Tumor‐recruited M2 macrophages promote gastric and breast cancer metastasis via M2 macrophage‐secreted CHI3L1 protein. J Hematol Oncol. 2017;10(1):36. 28143526 10.1186/s13045-017-0408-0 PMC5286803

[27] Libreros S , Garcia‐Areas R , Shibata Y , Carrio R , Torroella‐Kouri M , Iragavarapu‐Charyulu V . Induction of proinflammatory mediators by CHI3L1 is reduced by chitin treatment: decreased tumor metastasis in a breast cancer model. Int J Cancer. 2012;131(2):377‐386. 21866546 10.1002/ijc.26379 PMC3288379

[28] Ma K , Yang L , Shen R , et al. Th17 cells regulate the production of CXCL1 in breast cancer. Int Immunopharmacol. 2018;56:320‐329. 29438938 10.1016/j.intimp.2018.01.026

[29] Godoy P , Cadenas C , Hellwig B , et al. Interferon‐inducible guanylate binding protein (GBP2) is associated with better prognosis in breast cancer and indicates an efficient T cell response. Breast Cancer. 2014;21(4):491‐499. 23001506 10.1007/s12282-012-0404-8

[30] Sekine T , Perez‐Potti A , Nguyen S , et al. TOX is expressed by exhausted and polyfunctional human effector memory CD8(+) T cells. Sci Immunol. 2020;5(49). 10.1126/sciimmunol.aba7918 32620560

[31] Kim K , Park S , Park SY , et al. Single‐cell transcriptome analysis reveals TOX as a promoting factor for T cell exhaustion and a predictor for anti‐PD‐1 responses in human cancer. Genome Med. 2020;12(1):22. 32111241 10.1186/s13073-020-00722-9 PMC7048139

[32] Buus R , Sestak I , Kronenwett R , et al. Molecular drivers of Oncotype DX, Prosigna, EndoPredict, and the Breast Cancer Index: a transATAC study. J Clin Oncol. 2020:Jco2000853. 10.1200/JCO.20.00853 PMC8078458 33108242

[33] Wallden B , Storhoff J , Nielsen T , et al. Development and verification of the PAM50‐based Prosigna breast cancer gene signature assay. BMC Med Genomics. 2015;8:54. 26297356 10.1186/s12920-015-0129-6 PMC4546262

[34] Ma XJ , Salunga R , Dahiya S , et al. A five‐gene molecular grade index and HOXB13:IL17BR are complementary prognostic factors in early stage breast cancer. Clin Cancer Res. 2008;14(9):2601‐2608. 18451222 10.1158/1078-0432.CCR-07-5026

[35] Filipits M , Rudas M , Jakesz R , et al. A new molecular predictor of distant recurrence in ER‐positive, HER2‐negative breast cancer adds independent information to conventional clinical risk factors. Clin Cancer Res. 2011;17(18):6012‐6020. 21807638 10.1158/1078-0432.CCR-11-0926

[36] Cardoso F , van't Veer LJ , Bogaerts J , et al. 70‐gene signature as an aid to treatment decisions in early‐stage breast cancer. N Engl J Med. 2016;375(8):717‐729. 27557300 10.1056/NEJMoa1602253

