# Supplementary material for: Correlation of immune infiltration with clinical outcomes in breast cancer patients: The 25‐gene prognostic signatures model
Source: Cancer Med. 2021 Feb 24;10(6):2112–24. doi: 10.1002/cam4.3678 (PMC7957182; doi:10.1002/cam4.3678)
Supplement: Supplementary file 1 — Table S1 [file CAM4-10-2112-s001.docx]

**Supplementary**

**Table 1.** Internal validation

| **Internal validation cohort(n=100)** | **Median risk score** | **Kaplan-Meier survival analysis** | **AUC values of 3-years** | **AUC values of 5-years** | **AUC values of 10-years** |
| --- | --- | --- | --- | --- | --- |
| 1 | -1.18 | p = 0.00037 | 0.93 | 0.946 | 0.872 |
| 2 | -1.19 | p = 0.029 | 0.775 | 0.851 | 0.944 |
| 3 | -1.11 | p = 0.0035 | 0.800 | 0.796 | 0.707 |
| 4 | -1.16 | p = 0.018 | 0.852 | 0.642 | 0.709 |
| 5 | -1.15 | p = 0.031 | 0.813 | 0.82 | 0.648 |
| 6 | -1.17 | p = 0.0063 | 0.762 | 0.791 | 0.849 |
| 7 | -1.12 | p = 0.017 | 0.858 | 0.696 | 0.719 |
| 8 | -1.07 | p = 0.00022 | 0.693 | 0.715 | 0.766 |
| 9 | -1.13 | p = 0.0022 | 0.708 | 0.837 | 0.874 |
| 10 | -1.06 | p = 0.00034 | 0.948 | 0.848 | 0.809 |
| 11 | -1.19 | p = 0.21 | / | / | / |
| 12 | -1.18 | p = 0.0015 | 0.861 | 0.900 | 0.765 |

p<0.05 was considered statistically significant
